# Lifestyle Attitudes and Habits in a Case Series of Patients With Cancer and Metabolic Syndrome

**DOI:** 10.1177/15598276251319262

**Published:** 2025-02-07

**Authors:** Kimberly Siu, Isabel Martinez Leal, Natalia I. Heredia, Jessica T. Foreman, Jessica P. Hwang

**Affiliations:** 1 Department of General Internal Medicine, 4002 The University of Texas MD Anderson Cancer Center, Houston, TX, USA (KS, JTF, JPH); 2 Department of Behavioral Science, 4002 The University of Texas MD Anderson Cancer Center, Houston, TX, USA (IML); 3 Department of Health Promotion and Behavioral Sciences, 49219 The University of Texas Health Science Center at Houston School of Public Health, Houston, TX, USA (NIH)

**Keywords:** cancer, metabolic syndrome, lifestyle changes, attitudes

## Abstract

Metabolic syndrome (MetS) is associated with increased cancer risk and poor cancer prognosis. The current study applied a mixed methods approach to better understand attitudes about making lifestyle changes and current dietary and physical activity behaviors among cancer patients with MetS at a cancer center and to explore the suitability of brief lifestyle questionnaires to help providers understand their patients’ lifestyle attitudes and habits. Qualitative interviews were used to obtain patients’ perspectives about lifestyle changes, and 3 quantitative questionnaires—the Readiness Ruler, Rate Your Plate, and the International Physical Activity Questionnaire-Short Form (IPAQ-SF)—were used to measure patients’ readiness for lifestyle change, dietary habits, and physical activity levels, respectively. Nineteen patients participated in interviews, and 18 patients completed the questionnaires. Interview findings indicated that patients generally prioritized lifestyle changes over medication use, desired collaboration and coordination between multidisciplinary care teams and patient, and desired tailored interventions and practical implementation strategies to manage MetS. Questionnaire findings indicated that most patients agreed with the importance of lifestyle changes and expressed confidence in making them, reporting healthy food choices and high physical activity levels. A multidisciplinary approach tailored to patients’ readiness, preferences, and constraints is recommended for effective MetS management in patients with cancer.

“Primary care providers wishing to promote positive lifestyle change in cancer patients with MetS would benefit from adopting a multidisciplinary approach.”

## Introduction

Metabolic syndrome (MetS) is characterized by hypertension, dyslipidemia, hyperglycemia, and increased waist circumference.^1^ Research links MetS to increased cancer risk and poor cancer prognosis.^2-6^ Currently, there is no clear guidance for medical providers on managing MetS in patients with cancer.

Lifestyle behaviors influence the risk and severity of MetS. As such, medical providers need to understand patients’ attitudes about lifestyle changes and willingness to engage in lifestyle interventions to empower patients to share considerations of lifestyle treatment options.^7^ Given time constraints during clinical encounters, it would be helpful if such lifestyle information could be obtained in advance of visits.

The purpose of the current mixed methods study was to better understand attitudes about lifestyle changes and current dietary and physical activity behaviors among cancer patients with MetS by using interviews and lifestyle questionnaires. Patients with cancer receiving anticancer therapies experience physical and psychological burdens that could limit their motivation to make lifestyle changes.^8^ Therefore, understanding cancer patients’ attitudes and concerns is vital to addressing the challenges to lifestyle change specific to this population. We also explored the suitability of brief lifestyle questionnaires to help providers understand their patients’ lifestyle attitudes and habits.

## Methods

### Study Design

The study was reviewed and approved by the Institutional Review Board of our institution. All patients gave informed consent before participation. We used pseudonyms to further protect their identity. All patients had cancer and MetS and received care for their MetS at our institution. Patients were interviewed and completed brief lifestyle questionnaires, as detailed below. The thematic analysis of the interviews was recently reported.^9^ Participant recruitment and eligibility are also detailed in the previous report.^9^ Demographic, clinical, and laboratory information was collected from electronic medical records. Liver ultrasonography with elastography was performed to assess for liver steatosis and fibrosis.

### Interviews

After providing informed consent, 19 patients engaged in interviews lasting approximately 60 min conducted online between June 2022 and February 2023 by 3 team members using a semistructured interview guide.^9^ Interviews were audio-recorded and transcribed verbatim. Interview questions focused on patients’ knowledge about and attitudes towards MetS, patient-identified barriers to and facilitators of adopting lifestyle changes, and needs and preferences for future interventions. Details on qualitative data collection are reported elsewhere.^9^

### Questionnaires

All participants were asked to complete 3 lifestyle questionnaires electronically.

The Readiness Ruler^10^ is a tool used in Motivational Interviewing and assesses readiness to make lifestyle changes through 2 questions: (1) How important is lifestyle change to you right now? And (2) How confident are you about making lifestyle changes? Possible answers range from 0 to 10, with 0-1 indicating *not very important/confident*, 2-8 indicating *somewhat important/confident*, and 9-10 indicating *very important/confident*.

Rate Your Plate^11^ is a 24-item food frequency questionnaire that asks about consumption levels of different foods and food groups, mealtime preferences and practices, and dining out. Points for all 24 items are summed to generate a score; higher scores indicate more healthy eating habits. A score of 24-40 points indicates *there are many ways you can make your eating habits healthier*, 41-57 indicates *there are some ways you can make your eating habits healthier*, and 58-72 indicates *you are making many healthy choices*.

The International Physical Activity Questionnaire—Short Form (IPAQ-SF)^12^ assesses physical activity levels using 7 items. Patients are asked to report their physical activity in the last 7 days, including sitting, walking, and moderate and vigorous physical activities, and are assigned to 1 of 3 physical activity levels^13^: (1) *low*, defined as physical activity less than the following 2 categories; (2) *moderate*, defined as at least 20 min per day of vigorous activity during 3 or more days, or at least 30 min per day of moderate activity during 5 or more days, or any combination of activity during 5 or more days achieving at least 600 metabolic equivalent task-minutes per week; and (3) *high*, defined as 3 or more days of vigorous physical activity, or any combination of activity during 7 or more days achieving at least 3000 metabolic equivalent task-minutes per week.

### Data Analysis

Clinical and laboratory data were analyzed using clinically significant thresholds. We define MetS according to the American Heart Association/National Heart, Lung, and Blood Institute guidelines, which include hypertension, dyslipidemia, hyperglycemia, and increased waist circumference.^1^ Although that guideline uses waist circumference as a criterion for diagnosis of MetS, we used body mass index (BMI), as BMI was a standard measurement in our institution during the study period but waist circumference was not. Overweight was defined as BMI ≥25 kg/m^2^, and obesity was defined as BMI ≥30 kg/m^2^.^14^ For Asian adults, thresholds for overweight and obesity were 23 kg/m^2^ and 25 kg/m^2^, respectively.^15^ High-density lipoprotein (HDL) levels were defined as normal if ≥40 mg/dL for males and ≥50 mg/dL for females.^1^ Triglyceride levels were defined as high if ≥150 mg/dL.^1^ Blood pressure was defined as high if systolic blood pressure was ≥130 mmHg and/or diastolic blood pressure was ≥85 mmHg.^1^

We also measured liver fibrosis and liver stiffness. Increased risk of liver fibrosis was indicated by a Fibrosis-4 (FIB-4) score ≥1.3.^16,17^ Liver stiffness ≥1.7 m/s on liver ultrasonography with elastography indicated a risk of compensated advanced chronic liver disease.^18^

Methods for qualitative thematic analysis of interviews are detailed elsewhere.^9^ Findings on qualitative interviews and lifestyle questionnaires were examined to explore patients’ perspectives about making lifestyle changes and their current dietary and physical activity behaviors. All analyses were organized using Dedoose (version 9.0.90, Los Angeles, CA: Sociocultural Research Consultants, LLC).

## Results

### Patient Characteristics

Nineteen patients met the inclusion criteria and enrolled. Among these patients, 11 (58%) had obesity, and 6 (32%) were overweight; the mean BMI was 32.8 kg/m^2^. All patients except 1 had normal HDL levels (n = 18, 95%). 10 patients (53%) had high triglyceride levels. Thirteen patients (68%) had high blood pressure. Seven patients (37%) had HbA1c level ≥6.5%. Mean FIB-4 score was 1.5; 11 patients (58%) had a FIB-4 score ≥1.3. Median ALT level was 23 U/L (range, 11-39 U/L). There were 17 patients who had liver imaging; 16 patients had liver ultrasound with elastography, and 1 had liver ultrasound without elastography. Among the 16 patients who had liver ultrasound with elastography, 14 had steatotic liver disease, 3 had liver stiffness ≥1.7 m/s, and 2 had liver masses/abnormalities. For the patient who had liver ultrasound without elastography, though fibrosis was not able to be detected without elastography, steatosis and a liver mass/abnormality were noted.

### Thematic Analysis of Qualitative Interviews

Analysis of the interviews yielded 5 themes: (1) understanding of MetS—patients had limited knowledge but desired education about this condition; (2) attitudes and approaches to MetS—motivation and prioritization of lifestyle changes were highly valued over medication use; (3) capacity and limitations in managing MetS—lifestyle change facilitators included family support, and barriers involved financial limitations and cancer-related issues; (4) patient-led care—with communication, collaboration, and coordination between the medical team and patient; and (5) co-creation of tailored intervention plans—patients desired instructions, practical guidance, and regularly monitored treatment plans based on and adjusted to their needs, resources, and support. Table 1 displays exemplary interview quotations that underpin these qualitative themes and correspond to questionnaire responses, along with recommendations for providers in managing MetS.

**Table 1.** Joint Display of Quantitative and Qualitative Findings From a Study of Cancer Patients With Metabolic Syndrome (MetS) About Their Attitudes Toward Making Lifestyle Changes and Current Dietary and Physical Activity Habits, Along With Corresponding Recommendations for Providers.

Attitudes and Habits	Responses to Quantitative Questionnaires		Selected Quotations From Qualitative Interviews	Recommendations for Providers
Readiness ruler (N = 18)				
*Attitude*: *Importance of making lifestyle changes*	*Lifestyle change is __ important*.	Very (8)	*“I’d rather do the work and try to get my weight down…because…I did gain a lot of weight*. *She gave me different ideas of what to do*, *exercising and even got me with a nutritionist*. *I changed my eating habits*, *and I started exercising*, *and I am down 18 pounds…”* (Parker, 58 years old, head and neck cancer) Theme: Attitudes and approaches to managing MetS	**Leverage** patients’ commitment to making lifestyle changes by offering comprehensive strategies to meet their nutritional and physical activity goals.
		Somewhat (9) or not very (1)	*“Let me put this in context*. *I’m really not worried about metabolic syndrome*; *my chief concern is treating the cancer*. *Something’s going to kill me*. *I’m 84 years old*, *and this is an end game*.*”* (Taylor, 84 years old, genitourinary cancer) Theme: Understanding of MetS	**Empathize **with patients who are currently not open to making lifestyle modifications about their overall priorities and what matters most to them.
*Attitude*: *Confidence in making lifestyle changes*	*I am __ confident in making lifestyle changes*.	Very (9)	*“It’s been the team together that has helped me feel like I could ask the questions that I needed and get the education and support that I needed to do what I needed to do*. *That’s very good*. *Allowing the patient to have the resources that they need to take control*. *I’m gonna do this…”* (Ryan, 67 years old, gynecologic cancer) Theme: Patient-led care	**Collaborate** with patients and other providers on their care team to co-create personalized interventions that build on patients’ internal motivation and foster patient autonomy.
		Somewhat (8) or not very (1)	*“Right now*, *I would say it’s more economics*, *like everything’s getting more expensive*. *Right now*, *it’s like*, *you cannot really pick what you want*, *like organic this or organic that*, *so that would be a challenge*.*”* (Kai, 57 years old, gynecologic cancer) Theme: Capacity and limitations in managing MetS	**Offer** resources to help overcome barriers to making lifestyle changes, such as social programs for financial limitations and dietitians for nutrition education.
Rate your plate (N = 18)				
*Habit*: *Current dietary behaviors*	*I am making many healthy choices*. (10)		*“Well*, *I think that they’ve been very helpful as far as letting me know what the goals are as far as my fruits and my vegetable intake*. *Now*, *I don’t do any red meats*. *Very rarely do I do–I do some organic chicken sometimes and two kinds of wild-caught fish*, *but mostly I’ve been concentrating on doing more of the vegan diet*. *I think it’s also being there*, *like when you have a question…Just questions on even though I’m eating healthy for health-wise is it okay for me for my cancer-wise…”* (Ryan, 67 years old, gynecologic cancer) Theme: Co-creation of tailored intervention plans	**Reassure** patients who are already making lifestyle changes that resources and support are available to address any questions that may come up during cancer treatment and to help sustain their healthy eating habits.
	*There are some ways* (7) *or many ways* (1) *to improve my diet*.		*“My problem*, *now*, *is not that—I’m never hungry*. *Most of the times*, *I make myself eat because I take medication*, *and bein’ a cancer patient*, *I need to keep weight on*. *I try to eat even when I really don’t want to*. *The food tastes—still tastes good*. *It’s just my appetite*. *I really don’t want it*. *I don’t have a weight problem*. *I have a problem with keepin’ the weight I have on*.*”* (Riley, 68 years old, lung cancer) Theme: Capacity and limitations in managing MetS	**Refer** patients who are struggling with dietary goals to dietitians who can help incorporate familiar foods while considering cancer-related challenges like decreased appetite and taste.
International physical activity questionnaire—short form (N = 18)				
*Habit*: *Current physical activity* *Behaviors*^a^	*Low physical activity level* (3)		*“I’ll be real honest with you*. *I’m super busy and I’ve got two super busy kids*, *so honestly*, *I—it was kind of more of a passing*, *‘You know*, *I should get out and walk’*, *or*, *‘I should get out and do something’*, *and then…I was diagnosed with cancer…I was still healing from surgery…I was getting chemo…This is a wake-up call for me*. *I need to take better care of myself… and so I’ve been trying…”* (Sam, 44 years old, central nervous system cancer) Theme: Co-creation of tailored intervention plans	**Acknowledge** the challenges to making changes to physical activity and consider simple strategies like the American College of Lifestyle Medicine cancer toolkit^19^ to help meet patients where they are regarding cancer-related mobility issues, if appropriate.
	*High physical activity level* (15)		*“Well*, *I don’t think that the … team…believed when I told them that…I could actually walk fast enough to do myself any good*. *She mentioned strolling*. *I told her I was not strolling*, *I was walking as fast as I could walk*. *Now I know that I am walking just as fast in my neighborhood as I am walking on the indoor track at the gym*, *or on the treadmill*. *Now*, *I hope to get better*, *but I’m at a 20-min mile right now*.” (Alex, 72 years old, gynecologic cancer) Theme: Patient-led care	**Commend** patients for making physical activity a habit and encourage them to maintain their routines, leveraging their resources for support.

^a^There were no patients in our study that reported a moderate physical activity level.

### Joint Analysis of Questionnaire Responses and Corresponding Interview Themes

Eighteen patients completed the lifestyle questionnaires. One patient did not respond to multiple requests to complete the questionnaires.

#### Readiness Ruler

On the Readiness Ruler, lifestyle changes were rated as *very important* by 8 patients, *somewhat important* (scores ranged 5-8, see Supplemental Table) by 9, and *not very important* by 1. Patients who believed lifestyle changes were *very important* were more likely to be receptive to education on MetS and report that lifestyle change was a priority during interviews.

In terms of making lifestyle changes, 9 patients felt *very confident*, 8 felt *somewhat confident* (scores ranged 4-8, see Supplemental Table), and 1 felt *not very confident*. Most of the participants who felt *very confident* or *somewhat confident* about making lifestyle changes also described during interviews viewing the cancer diagnosis as a wake-up call, feeling motivated to change lifestyle, and reaching their potential and goals.

Most of the patients who felt *very confident* about making lifestyle changes and most of the patients who believed lifestyle changes were *very important* also requested a collaborative and coordinated approach from their medical team that centered on their needs, preferences, and autonomy.

#### Rate Your Plate

On the Rate Your Plate questionnaire, 10 patients had scores indicating that they were making many healthy food choices, 7 had scores indicating that they had some ways to make eating habits healthier, and 1 had a score indicating that the patient had many ways to make eating habits healthier. Compared with patients with some or many ways to improve their diet, patients making many healthy food choices were more likely to request additional education about MetS, consider lifestyle as a priority, and reach self-actualization about making lifestyle changes. Interestingly, patients making many healthy food choices often reported financial barriers.

Participants with some or many ways to improve their diet reported limitations to adopting a healthier lifestyle, such as physical constraints (e.g., side effects from cancer treatment), time constraints due to family and work responsibilities, and existing family food preferences. These patients also asked to participate in making decisions about intervention plans designed via collaboration and coordination between the medical team and patient.

#### International Physical Activity Questionnaire—Short Form

On the IPAQ-SF, 15 patients reported engaging in high physical activity, no patients reported moderate physical activity, and 3 patients reported low physical activity. Interestingly, many patients who reported engaging in high physical activity expressed during interviews difficulties with adopting healthier habits due to side effects of cancer treatment (e.g., peripheral neuropathy).

## Discussion

In this mixed methods study of 18 cancer patients with MetS, interviews revealed that patients had limited knowledge of MetS, valued lifestyle change over medication use, and desired to collaborate with medical providers in developing tailored intervention plans. The interviews also revealed facilitators of and barriers to adoption of lifestyle changes. In responses to brief lifestyle questionnaires, 8 patients indicated that lifestyle change was very important, 9 indicated that they were very confident in making lifestyle changes, 10 reported making many healthy food choices, and 15 reported engaging in high physical activity. Our findings suggest brief lifestyle questionnaires may help medical providers understand their patients’ lifestyle attitudes and habits to guide effective discussions about tailored interventions to treat MetS.

Patients who revealed intrinsic senses of urgency, self-efficacy, and motivation to incorporate healthy habits into one’s lifestyle during interviews had favorable scores on the Readiness Ruler and Rate Your Plate questionnaires. For patients with high readiness to change, providers could collaborate with other members of the care team to develop personalized, rigorous, and comprehensive lifestyle interventions. Such lifestyle interventions might include clear and actionable instructions to facilitate tailored lifestyle change that would be meaningful to each patient, such as healthy recipes for cooking nutrient-dense meals, instructions for specific physical exercises, and guidance for dining out, along with frequent check-ins. Adoption of healthy habits may have indicated successful navigation of barriers to lifestyle change among patients with high readiness. Future research could inform interventions aimed at supporting patients in identifying and addressing barriers to healthy habits.

Patients with unfavorable Readiness Ruler and Rate Your Plate scores may have competing concerns unknown to their care team. Rather than developing lifestyle intervention plans immediately, providers could first consider asking these patients about their overall priorities and goals. Motivational Interviewing techniques could be a useful communication strategy to initiate conversations about personal change with patients of low readiness. Our findings suggest that creating a compassionate space for patients to voice their concerns and needs could be a valuable starting point to align perspectives to foster effective care and balance the goal of avoiding unintentional weight loss with the goal of maintaining a healthy lifestyle. A patient’s hesitation about or indifference to lifestyle change may signal a desire for more connection about other concerns in their lives. Guiding patients to the American College of Lifestyle Medicine Cancer Toolkit might be an ideal strategy to introduce concepts that align with wherever the patients are in their cancer journey.^19^ Using the general framework and principles of behavioral change models such as the transtheoretical stages of change and the ecological models of health behavior, providers could assess where patients are in their journey of lifestyle change and address patients appropriately.^20-23^ Providers could also inquire about physical and social limitations, particularly with patients who demonstrate moderate readiness and some healthy habits, as such patients may be attempting to change but be hindered by social or financial obstacles.

Our study has some limitations. First, BMI is a flawed measure of body fat in individuals.^24^ However, during the study period, BMI was a standard measurement used in our institution to help identify patients with metabolic syndrome, and waist circumference was not routinely collected. Other measurements may be more useful for other populations.^25,26^ Second, IPAQ-SF scores in our series of patients with cancer were unexpectedly high and discordant with interview responses. Participants who reported high physical activity on the IPAQ-SF highlighted difficulties with physical activity due to cancer treatment during interviews. While not explored in-depth during qualitative interviews, it is possible that these participants may have previously navigated these barriers during their cancer journey and were subsequently able to achieve high physical activity in the past 7 days. On the other hand, the discordance between IPAQ-SF score and interview responses may be a result of overestimated physical activity levels.^27^ While the IPAQ-SF is a cost-effective, simple, and minimally burdensome tool that captures diverse physical activity domains, the IPAQ-SF may not be an ideal questionnaire to assess physical activity in patients with cancer, and future studies may consider using other brief measures, such as the Physical Activity Vital Sign.^28^

Our findings suggest that primary care providers wishing to promote positive lifestyle change in cancer patients with MetS would benefit from adopting a multidisciplinary approach that facilitates cooperation and communication between medical providers and patients before proceeding with specific lifestyle interventions. Collaboration among primary care and oncology providers, physical therapists, and registered dietitian nutritionists may be useful for understanding patients’ ongoing medical concerns and addressing side effects of cancer treatment and other barriers to lifestyle interventions. These collaborative steps may enhance informed decision-making among patients, encouraging readiness for lifestyle changes tailored to their perspectives, willingness, and capacities. Future studies that explore strategies to address complications of cancer treatment by offering modified lifestyle interventions will support patients with cancer and metabolic syndrome.

## Supplemental Material


Supplemental Material - Lifestyle Attitudes and Habits in a Case Series of Patients With Cancer and Metabolic Syndrome
Supplemental Material for Lifestyle Attitudes and Habits in a Case Series of Patients With Cancer and Metabolic Syndrome by Kimberly Siu, Isabel Martinez Leal, Natalia I. Heredia, Jessica T. Foreman and Jessica P. Hwang in American Journal of Lifestyle Medicine.
