# Supplementary material for: Lifestyle Attitudes and Habits in a Case Series of Patients With Cancer and Metabolic Syndrome
Source: Am J Lifestyle Med. 2025 Feb 7;20(5):760–8. doi: 10.1177/15598276251319262 (PMC11806445; doi:10.1177/15598276251319262)
Supplement: Supplemental Material - Lifestyle Attitudes and Habits in a Case Series of Patients With Cancer and Metabolic Syndrome [file sj-pdf-1-ajl-10.1177_15598276251319262.pdf]

Supplemental Table. Readiness Ruler responses by participant

Participant	Readiness Ruler	
	**Importance of Lifestyle Change**	**Confidence in Making Lifestyle Change**
1	10 (Very important)	10 (Very confident)
2	6 (Somewhat important)	7 (Somewhat confident)
3	10 (Very important)	6 (Somewhat confident)
4	8 (Somewhat important)	8 (Somewhat confident)
5	10 (Very important)	10 (Very confident)
6	8 (Somewhat important)	9 (Very confident)
7	10 (Very important)	10 (Very confident)
8	10 (Very important)	8 (Somewhat confident)
9	7 (Somewhat important)	9 (Very confident)
10	10 (Very important)	10 (Very confident)
11	5 (Somewhat important)	5 (Somewhat confident)
12	7 (Somewhat important)	9 (Very confident)
13	5 (Somewhat important)	5 (Somewhat confident)
14	1 (Not very important)	1 (Not very confident)
15	8 (Somewhat important)	8 (Somewhat confident)
16	10 (Very important)	10 (Very confident)
17	10 (Very important)	10 (Very confident)
18	5 (Somewhat important)	4 (Somewhat confident)
19	No response	No response
